# Phospholipase D3 (PLD3) Regulates Lysosomal Biogenesis

**DOI:** 10.1002/alz.087739

**Published:** 2025-01-03

**Authors:** Yongchao Wang, Wilber Romero Fernandez, Cristian O Carvajal‐Tapia, Alena Shostak, Paige Rieschick, Nathan Appelbaum, Matthew Schrag

**Affiliations:** ^1^ Vanderbilt University Medical Center, Nashville, TN USA; ^2^ Vanderbilt Brain Institute, Nashville, TN USA

## Abstract

**Background:**

Genome‐wide association studies suggest mutations in endolysosomal genes are linked to Alzheimer’s disease (AD). Defective lysosomal function has been corroborated as a feature of AD by neuropathological and cell biology studies. PLD3 is a homolog of the phospholipase D family localized to lysosomes. Genetic variants in PLD3 are associated with increased risks for AD. Nevertheless, being a resident lysosomal protein, PLD3’s role in lysosome homeostasis has not been completely characterized. In this study, we conducted studies that combine transcriptomics, proteomics and cell biology to investigate the role of PLD3 in lysosome homeostasis.

**Method:**

We generated PLD3 knockout (KO) SH‐SY5Y cells through the CRISPR/Cas9 approach. To identify the individual genes or gene sets impacted by PLD3 KO, RNA sequencing was performed with RNA isolated from wildtype (WT) and PLD3 KO neurons, followed by differential gene expression and gene set enrichment analyses. To narrow down to the targets that have direct lysosomal relevance, a proteomic study was conducted on the lysosome fraction of lysates from WT and PLD3 KO cells. To characterize lysosome morphology and function, lysosomes in these cells were labeled with Lysotracker or dextrans, followed by imaging, quantification and statistical analysis with CellInsight High Content Analysis platform. Lastly, we also examined the effects of PLD3 on lysosomal biogenesis by immunocytochemistry and Western blot.

**Result:**

In RNA‐Seq analysis, the genes or gene sets relevant to endocytosis, autophagy and lysosomal biogenesis were significantly enriched in PLD3 KO cells. In line with these findings, our proteomic analysis also demonstrated an increase in lysosomal proteins in PLD3 KO cells. PLD3 loss caused an increase in lysosome size and endocytic activity and a decrease in the pH value of lysosomes, but it had no significant impact on lysosome number and positioning. Mechanistically, PLD3 deficiency promoted TFEB‐mediated lysosomal biogenesis.

**Conclusion:**

PLD3 plays a role in lysosomal biogenesis. As endolysosomal abnormalities are increasingly appreciated as the causal agent for the pathogenesis of AD, this study may position the PLD3 pathway as a therapeutic target for the treatment of AD.

